# TRPV1 and Piezo: the 2021 Nobel Prize in Physiology or Medicine

**DOI:** 10.1107/S2052252521013488

**Published:** 2021-12-23

**Authors:** Yifan Cheng

**Affiliations:** a Department of Biochemistry and Biophysics, and Howard Hughes Medical Institute, University of California San Francisco, CA, USA

**Keywords:** TRPV1, Piezo, nociception, Nobel Prize

## Abstract

The 2021 Nobel Prize in Physiology or Medicine honors the discoveries of the temperature or mechanically activated channels, whose structural studies provided insights of channel gating at atomic level.

The Royal Swedish Academy of Sciences awarded the 2021 Nobel Prize in Physiology or Medicine to David Julius and Ardem Patapoutian for ‘their discoveries of receptors for temperature and touch’. Using capsaicin, a natural pungent agent that induces pain in our skin similar to that induced by heat, David Julius identified the TRPV1 ion channel as a heat-activated nociceptor in the peripheral nervous terminus (Caterina *et al.*, 1997 ▸). By identifying a mouse cell line that generates robust mechanically activated cationic ion channel currents, Ardem Patapoutian discovered Piezo1 and Piezo2, long-sought mechanically activated cation channels in vertebrates including mammals (Coste *et al.*, 2010 ▸). David Julius and Ardem Patapoutian also independently discovered TRPM8 as the cold-activated nociceptor (McKemy *et al.*, 2002 ▸; Peier *et al.*, 2002 ▸).

TRPV1, TRPM8 and Piezo1/2 are all ion channels in the plasma membrane of neuronal cells that can be activated in response to stimuli such as temperature, chemicals and mechanical force. Upon stimulation, these channels open to allow an influx of cations to depolarize the cell membrane and generate action potentials, which transmits signals along the neuron to the spinal cord. Discoveries of these nociceptors or mechanosensors triggered a wide range of physiological, biochemical and biophysical studies of these sensory ion channels, and consequently advanced and broadened our understanding of how the human body senses the environmental, internal noxious stimuli or gentle touch.

The structural study of these ion channels is also an important yet very challenging part of the functional and physiological investigation of nociception. Such efforts often start with determining the atomic structures of these ion channels, followed by capturing their structures at different functional states, including those activated by external and internal stimuli. The availability of known ligands that can either activate or inhibit channel function provides valuable tools to facilitate capturing structures in these different functional states. Such structural biology efforts aim to provide a comprehensive mechanistic understanding of how these channels behave in response to stimuli and other modulators.

Early successful examples of structural studies of ion channels by X-ray crystallography include potassium channels, which was recognized by the 2003 Nobel Prize in Chemistry. Similarly, the discoveries of TRPV1, TRPM8 and Piezo1/2 also sparked intensive efforts from the structural biology community to determine the structures of these channels and to uncover the mechanisms by which these channels respond to stimuli. However, TRPV1 and TRPM8, as well as all other members of the TRP channel superfamily, resisted crystallization despite tremendous efforts from crystallographers around the world. Thus, the structure of both TRPV1 and TRPM8 remained unknown for a long time. (The discovery of Piezo1/2 came after TRPV1 and TRPM8, thus its structure remained unknown for a relatively short period of time.) Unexpectedly, the atomic structures of these channels were all determined by single-particle cryo-electron microscopy (cryo-EM), a structure determination technique that does not require crystallization but, instead, images individual biological molecules embedded in a thin layer of vitreous ice using an electron microscope followed by extensive computational image analysis (Cheng, 2018 ▸).

Interestingly, progress in structural studies of TRPV1 and the technological developments and advancements of single-particle cryo-EM were tightly entwined. With the methodological breakthroughs in single-particle cryo-EM, the first structure of TRPV1 was determined in 2013 (Cao *et al.*, 2013 ▸; Liao *et al.*, 2013 ▸). Remarkably, this first atomic structure of a membrane protein determined by this method without crystallization energized the structural biology community, triggered the so-called ‘resolution revolution’, and attracted major efforts to advance the methodology further. Single-particle cryo-EM has now changed the landscape of structural biology (Callaway, 2015 ▸Subramaniam *et al.*, 2016 ▸), and was recognized by the 2017 Nobel Prize in Chemistry. For TRPV1, the continuous methodological advancements in single-particle cryo-EM has not only deepened our mechanistic understanding of channel gating, but also raised new questions that often require further methodology development. Such intertwined paths of technological development and biological discoveries expanded our knowledge of how TRPV1 is regulated in response to noxious stimuli.

As a polymodal signal detector, TRPV1 can be activated by exogenous stimuli, such as heat, capsaicin, plant toxins and peptide toxins from spider *etc*. These exogenous stimuli generate pain that serve as a warning signal for avoidance. TRPV1 activation can also be modulated by endogenous stimuli, such as extracellular protons generated by tissue acidosis associated with inflammation. Potentiation of TRPV1 by extracellular protons causes heat hypersensitivity. Without the restriction of crystallization, single-particle cryo-EM not only enabled atomic structure determination of TRPV1 (see Fig. 1 ▸, left panel) but also allowed the capture of channel structures in different functional states. These included the closed state when unliganded or in the presence of antagonists, and the fully open state when bound with single or multiple agonists. These structures now provide mechanistic insights at the atomic level into how the channel integrates numerous physiological stimuli (Cao *et al.*, 2013 ▸; Gao *et al.*, 2016 ▸; Zhang *et al.*, 2021 ▸).

As for Piezo1/2, soon after its initial discovery, structural biology had entered this new era of single-particle cryo-EM. As such, structural studies of Piezo channels were carried out exclusively using this method and comprehensive structural studies have progressed rather rapidly (Ge *et al.*, 2015 ▸; Wang *et al.*, 2019 ▸; Zhao *et al.*, 2018 ▸). The trimeric arrangement of a large transmembrane domain of the Piezo channels forms a three-bladed propeller-shaped structure that is unique among ion channels (see Fig. 1 ▸, right panel). The unusually curved shape of the Piezo channels revealed by their structures provides clues as to how the channel may be gated by sensing the membrane tension. While the lack of pharmacological tools to manipulate the channel to mimic mechanical activation may make it harder to fully reveal the mechanism of channel gating by mechanical forces, there are possible approaches that may allow the capture of the channel in mechano-activated open conformations.

The major goals of structural biology go beyond knowing the atomic structures of these channels and what they look like, but address the harder questions of how they work to generate physiological responses. In this regard, there are still many unanswered questions concerning these channels. Among them, the mechanisms of temperature activation, such as TRPV1 by heat or TRPM8 by cold, and the mechanism of mechanosensing by Piezos, remain among the most pressing unanswered questions. Recognition of the Nobel Prize not only honors the seminal discoveries of these channels, but also stimulates excitement among structural biologists to continue pursuing these burning questions.

## Figures and Tables

**Figure 1:**
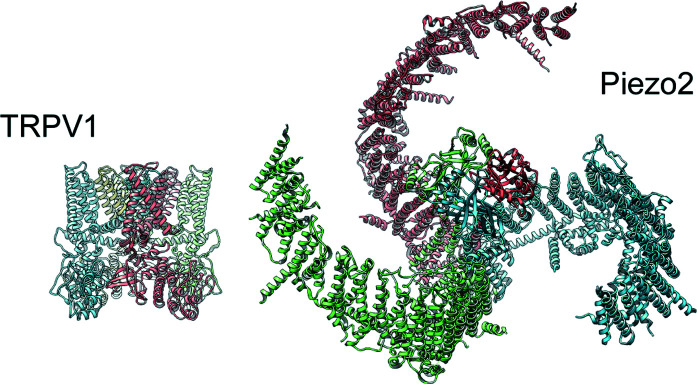
Structures of TRPV1 (left) and Piezo2 (right) represented in ribbon diagram. Piezo2 is slightly tilted around the horizontal axis to reveal its three-bladed propeller shape. Both structures were determined by single-particle cryo-EM.
